# Structural parameters for X-ray micro-computed tomography (μCT) and their relationship with the breakage rate of maize varieties

**DOI:** 10.1186/s13007-019-0538-1

**Published:** 2019-12-27

**Authors:** Junfeng Hou, Ying Zhang, Xiuliang Jin, Pengfei Dong, Yanan Guo, Keru Wang, Yinghu Fan, Shaokun Li

**Affiliations:** 1 Institute of Crop Science, Chinese Academy of Agricultural Sciences, Beijing, 100081 China; 2 Beijing Agricultural Information Technology Research Center, Beijing Key Laboratory of Digital Plants, Beijing, 1000973 China; 3 Agricultural College of Shihezi University, Shihezi, 832003 China; 4 Chuxiong State Research and Extension Institute of Agricultural Science, Chuxiong, 675000 China

**Keywords:** Breakage rate, X-ray μCT, Maize grain, Density, Subcutaneous cavity volume, Shape, Weight

## Abstract

**Background:**

High grain breakage rate is the main limiting factor encountered in the mechanical harvest of maize grain. X-ray micro-computed tomography (μCT) scanning technology could be used to obtain the three-dimensional structure of maize grain. Currently, the effect of maize grain structure on the grain breakage rate, determined using X-ray μCT scanning technology, has not been reported. Therefore, the objectives of this study are: (i) to obtain the shape, geometry, and structural parameters related to the breakage rate using X-ray μCT scanning technology; (ii) to explore relationships between these parameters and grain breakage rate.

**Result:**

In this study, 28 parameters were determined using X-ray μCT scanning technology. The maize breakage rate was mainly influenced by the grain specific surface area, subcutaneous cavity volume, sphericity, and density. In particular, the breakage rate was directly affected by the subcutaneous cavity volume and density. The maize variety with high density and low subcutaneous cavity volume had a low breakage rate. The specific surface area (r = 0.758*), embryo specific surface area (r = 0.927**), subcutaneous cavity volume ratio (0.581*), and subcutaneous cavity volume (0.589*) of maize grain significantly and positively correlated with breakage rate. The cavity specific surface area (− 0.628*) and grain density (− 0.934**) of maize grain significantly and negatively correlated with grain breakage rates. Grain shape (length, width, thickness, and aspect ratio) positively correlated with grain breakage rate but the correlation did not reach statistical significance. The susceptibility of grain breakage increased when kernel weight decreased (− 0.371), but the effect was not significant.

**Conclusions:**

The results indicate that X-ray μCT scanning technology could be effectively used to evaluate maize grain breakage rate. X-ray μCT scanning technology provided a more precise and comprehensive acquisition method to evaluate the shape, geometry, and structure of maize grain. Thus, data gained by X-ray μCT can be used as a guideline for breeding resistant breakage maize varieties. Grain density and subcutaneous cavity volume are two of the most important factors affecting grain breakage rate. Grain density, in particular, plays a vital role in grain breakage and this parameter can be used to predict the breakage rate of maize varieties.

## Background

Maize (*Zea mays* L.) is a vital global food source. Grain breakage is an important problem in maize harvesting, transportation, and processing. Breakage reduces the suitability of the grain for wet and dry milling and increases the risk of damage during storage [1]. The efficiency of dryer aeration systems may decrease if large proportions of kernels are broken, resulting in reduced yields and the production of toxic substances. At present, a high grain breakage rate is the leading problem in grain mechanical harvesting. Previous studies have shown that maize grain shape [1], internal structure, grain hardness [2], and chemical composition [3] affect grain breakage, and the sensitivity or resistance to grain breakage has high heritability [4–6]. Leford et al. [7] showed that breakage resistant grain tended to be smaller and denser, and was higher in shear strength. Waelti et al. [8] showed that moisture content and kernel size (thickness and area) were positively related to kernel damage; kernel damage increased with increasing kernel size and moisture content. Plant density, harvest moisture content, and drying temperature also influence grain breakage susceptibility [8]. Martin et al. [9] concluded that mechanical breakage at harvest was influenced primarily by kernel shape, size, and structural characteristics and then by kernel hardness properties. Exploring the grain characteristics related to grain breakage will facilitate the identification of key factors impacting grain breakage.

Breakage susceptibility is usually measured using the Stein breakage tester (SBT) and Wisconsin breakage tester (WBT) methods [9]. The SBT method uses a 100-g grain sample, which is impacted and abraded by continuous stirring with impeller rotation for 2 min. In the WBT method, a 200 g kernel sample is accelerated by centrifugal force and the impact on kernel breakage is determined. The degree of sphericity directly affects the fluidity of particles and represents the degree that objects are close to a sphere [9].

Kernel density could be measured using several approaches. The most commonly method is a bulk density value to evaluate maize quality. The floater test and pycnometer displacement test are more accurate methods for density measurements. The floater test is based on the percentage of suspended kernels in a given specific gravity salt solution. In the pycnometer measurement, water or gas is discharged from a large number of kernel samples, and then the density is calculated by dividing the mass of the sample by the volume discharged [10–12]. However, the material density of a single grain is difficult to obtain with these methods.

The traditional methods are commonly destructive and require sample preparation for obtaining shape, density, and the internal structure of kernels. In addition, the effects of grain surface area and volume on kernel breakage are difficult to evaluate. X-ray µCT can be used to easily acquire the geometric parameters of objects. X-ray µCT is non-destructive and acquires 3D imaging with resolutions higher than 1 µm, which allows the internal structural parameters of the sample to be analyzed [13]. X-ray µCT delivers unparalleled data with more detail than any other technique. The most compelling characteristics of the X-ray µCT techniques are digital imaging and 3D quantitative volume [14].

The X-ray µCT software tools system, Skyscan 1072 (Skyscan, Belgium), can easily acquire many parameters from 3D models, including air volume, surface-to-volume ratio, spatial cell size distribution, cell wall-thickness distribution, connectivity, and porosity. X-ray µCT technology, a useful method for studying the 3D structure of food material, can measure the density of a single grain [15, 16]. X-ray µCT has been successfully used for studying food, such as cream cheese [17], yogurt [18], and mayonnaise [19]. Donis-González et al. [20] and Kotwaliwale et al. [21] showed that the number of X-ray imaging appliances in agricultural research is increasing. The whole grain structure of high-amylose and wild-type rice were compared using X-ray µCT [22]. Van Dalen et al. [23] investigated porous cereal products and obtained the real density variations of a grain sample to exclude grain cavities using X-ray µCT, which is impossible using other imaging methods. X-ray μCT technology has also been used to quantify maize stem vascular bundles traits and metaxylem vessels in maize roots, to understand the relationship between root anatomy and function [24, 25]. The influence of grapevine xylem organization on the refilling of embolized vessels was discovered using High-resolution X-ray Computed Tomography (HRCT). Whole vessel network studies were helpful in comprehending how the distribution of interconnection affects hydraulic conductivity and the ability to adapt to the changing environment [26, 27]. Gustin et al. [16] obtained the parameters of maize grain density, volume, and cavity volume by X-ray µCT and demonstrated that the measured grain density significantly correlated with the bulk density (r = 0.80). Gustin et al. [16] also showed that embryos had little impact on kernel density and the ratio of vitreous endosperm/floury (V:F) endosperm showed a strong correlation with density. Guelpa [28] suggested that the ratio of vitreous endosperm/floury endosperm could be calculated using X-ray µCT to derive density and indicated a significantly higher V:F for the high-density kernel (2.77) compared to that of low-density kernels (1.27).

Based on previous studies, X-ray µCT scanning technology has rarely been used to acquire the 3D structure of maize grain. In addition, no reports have shown how to explore the effect of maize grain 3D structure on grain breakage rate using X-ray μCT scanning technology. Therefore, the objectives of this study were: (i) to obtain the shape (length, width, thickness, aspect ratio, and sphericity), geometry (the volume and proportion of the grain, endosperm, seed coat, embryo, and cavity and surface area), and structure (grain density and other characteristics) parameters that relate to the breakage rate using X-ray μCT scanning technology; (ii) to explore the relationships between these parameters and grain breakage rate. This study provides new insight into the relationship between grain breakage rate and grain structure information in maize.

## Materials and methods

### Material

The grains used in this study included Denghai 618 (DH618), Xianyu 335 (XY335), M751, KX3564, KX9384, and Lianchuang 808 (LC808). In 2017, the grains were planted with the same density in the same plot in Qitai, Xinjiang. A representative ear from each of the 6 varieties was naturally air-dried, and the grains were selected from the middle of each ear by manual threshing. Three replicates of the non-injured typical grains were used for test samples.

### Image acquisition and the distribution of grain components

The test samples were scanned using the Skyscan 1172 X-ray computed tomography system (Bruker Corporation) and a 1.3 megapixel cooled CCD camera. The source-to-object distance (the distance from the X-ray tube to the object) was 259.850 mm for all individuals and the source-to-image distance (the distance from the X-ray tube to the X-ray detector) was 345.591 mm. A 40 kV/250 mA tungsten X-ray source was employed for CT scan and the test samples scans were made over 180º rotations with the images taken every 0.2º. An exposure time of 1475 ms and two-frame averaging were chosen to produce images with a pixel size of 7 μm. The CT data in the 4 K scan module were converted into a series of CT slice images with 8-bit tagged image file format using Skyscan NRecon software (Bruker Corporation).

The kernel was placed on the translation stage with paraffin, and the bottom of the kernel was adhered with paraffin, which would not affect the level of X-ray attenuation. During scanning a sample was rotated on a translation stage while illuminated with X-rays. X-ray CT evaluated the internal structure of a sample by means of a X-ray source and a detector in order to obtain information from a projected slice. When an X-ray beam passed through a sample it was attenuated. The differences in attenuation were attributable to density and compositional differences within a sample. The X-rays passed through the object in many different directions and the transmission level was determined by the absorption coefficient of a sample. According to the degree of X-ray attenuation, the detector measured the remnant attenuated radiation and the response was transferred to a computer. An image was created along different pathways illustrating variation in density at numerous points in a 2D slice [15]. Thus, a series of 2D radiographs or projection images were acquired.

**Fig. 1:**
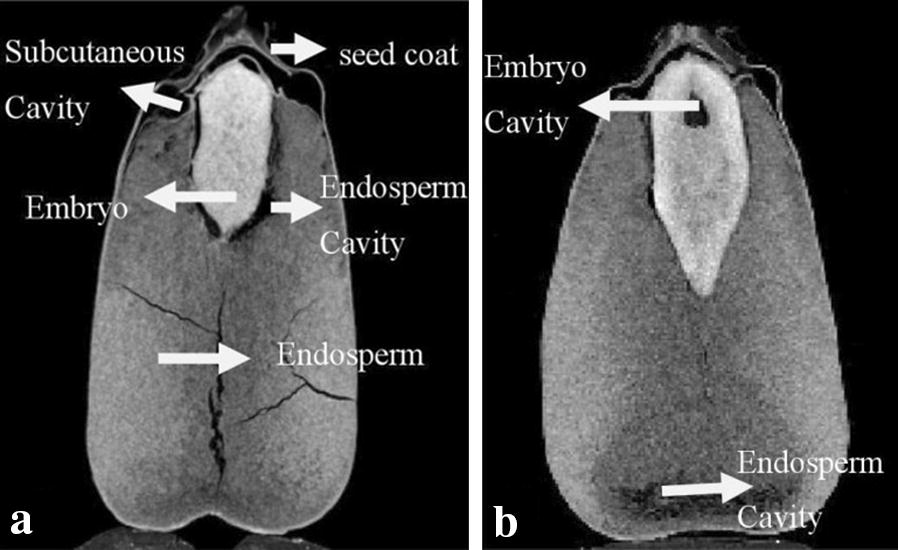
Components distribution of grain 2-D image. **a**, **b** show the different grain components, the brightest region is embryo, second region is endosperm, black region inside the grain is cavity. Different positions of cavity are separated into three parts, which are subcutaneous cavity, endosperm cavity and embryo cavity, respectively

**Fig. 2:**
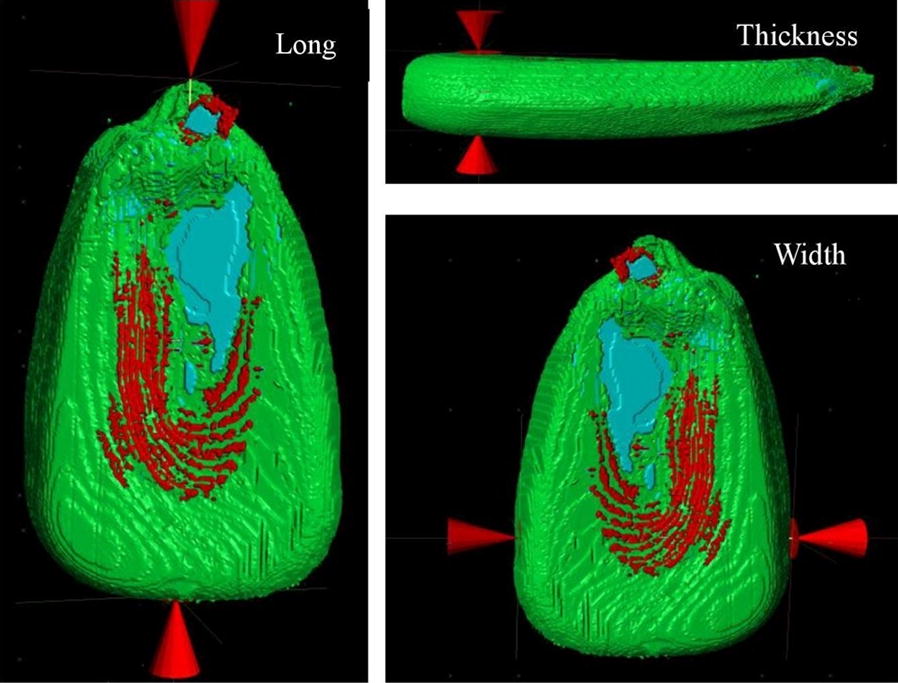
Three-dimensional measurement after the image reconstructed. The distance between the arrowheads is the dimension of length, width and thickness, respectively. Three colors represent the different parts of maize grain, green is endosperm, red is cavity space, blue is embryo

The entire grain spatial architecture was analyzed and visualized by CT-volume (CTVol) software (Bruker Corporation, Germany). Using 2D images from different angles of the grain sample, the internal structural information of different sections and three-dimensional reconstruction results were obtained (Figs. 1a–c and 2). The 2D images enable the visualization of the morphology and microstructure, such as the pore shape, size, and distribution. Grain 2D images were divided into four major parts: seed coat, embryo, endosperm, and cavity, which contained the subcutaneous cavity, endosperm cavity, and embryo cavity.

### Grain image reconstructed and quantitative analysis

**Fig. 3:**
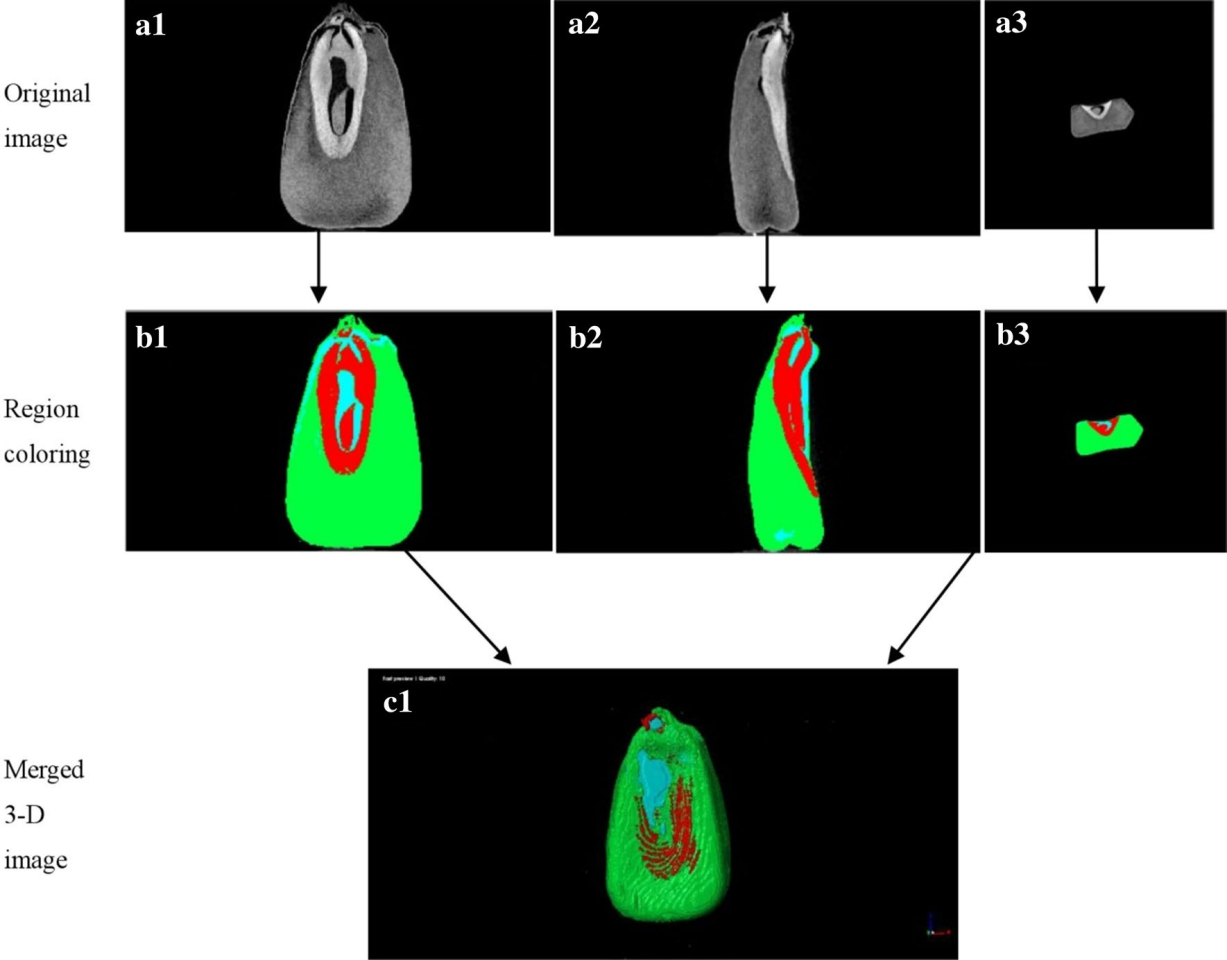
Image segmentation of the grain component. **a1**–**a3** are the three aspects of grain raw image, respectively; **b1**–**b3** are the region color segmentation; **c1** is the merged 3-D imagery. Three colors represent the different parts of maize grain, green is endosperm, red is cavity space, blue is embryo

The CT-Analyser (CTAn) and CT-Volume (CTVol) software were used to reconstruct CT raw images that were subsequently visualized in color. The image processing was carried out as described in the literature [29]. About 1000 images were obtained from the X-ray µCT of a single sample. The image analysis and segmentation processes applied include filter, adaptive thresholds, region-growing, erosion and dilation techniques, which were function key of the CTAn and CTVol software. First, filter was used to reduce noise and to correct detector defects. And filter was also applied to increase the visibility and to enhance the edges of a sample. Second, image segmentation was determined using processing techniques, including locally adaptive thresholds and region-growing techniques. Using locally adaptive thresholds, the voxel containing grey values lower or higher than this threshold value were regarded as background or sample material, respectively. 2D image was segmented depending on the brightness and regional distribution. Every image was divided into six parts, including the seed coat, embryo, endosperm, subcutaneous cavity, endosperm cavity, and embryo cavity (Fig. 1). Every part was dyed by different colors with region-growing, which could be done rely on the average grey value of every part. Third, after segmentation, the image was cleaned up to remove small quantities of pixels that could affect the result. The erosion and dilation tools were used for cleaning image. Fourth, the 2D slice images were merged to create a 3D image. Figure 3 showed the process of image segmentation.

Through the 3D image, the internal structure of the sample was visualized and the distribution of the different density regions was identified. The 3D image microstructural parameters, including volume, volume fractions, area, dimensions, and connectivity, along with the density information were obtained from the data sets. Using X-ray µCT, the geometry of structural components, such as cavity volume and ratio, were acquired from the raw data. The image processing was time-consuming and only two grain samples could be processed per day.

### Acquisition of grain morphology and structural parameters

Grain size, geometry, and structural parameters were determined by 3D X-ray µCT images. The geometry of grain components was quantified by size, shape, orientation, and position. We obtained 28 parameters related to grain shape and structure, which provided detailed data to understand maize kernels (Table 1). After the three-dimensional reconstruction of the maize kernel, the dimensions (length, width, and thickness) of the three-dimensional image were measured using the CTVol software. The weight was determined with an analytical balance.

**Table 1:** All parameters obtained through CT scan

Parameters	Features	Formulas or diagrams
Width (W)	The left to right distance of 3D image	Figure 2
Length (L)	The top to bottom distance of 3D image	Figure 2
Thickness (T)	Distance between embryo side and endosperm side of 3D image	Figure 2
Aspect ratio (AR)	The ratio of length to width	AR = L/W
Weight (mg)	The weight of single grain	–
Grain volume (Vg)	The whole volume of grain 3D image	–
Endosperm volume (Ven)	The whole volume of endosperm 3D image	–
Embryo volume (Vem)	The whole volume of embryo 3D image	–
Cavity volume (Vc)	The whole volume of cavity 3D image	–
Subcutaneous cavity volume (Vsc)	The volume of subcutaneous cavity	–
Embryo cavity volume (Vemc)	The volume of embryo cavity	–
Endosperm cavity volume (Venc)	The volume of endosperm cavity	–
Grain surface area (SAg)	The surface area of grain 3D image	–
Endosperm surface area (SAen)	The surface area of endosperm 3D image	–
Embryo surface area (SAem)	The surface area of embryo 3D image	–
Cavity surface area (SAc)	The surface area of cavity 3D image	–
Grain specific surface area (SSAg)	The ratio of grain surface to volume	SAg/Vg
Endosperm specific surface area (SSAen)	The ratio of endosperm surface to volume	SAen/Ven
Embryo specific surface area (SSAem)	The ratio of embryo surface to volume	SAem/Vem
Cavity specific surface area (SSAc)	The ratio of cavity surface to volume	SAc/Vc
Endosperm volume ratio (VRen)	The ratio of endosperm volume to grain volume	Ven/Vg
Embryo volume ratio (VRem)	The ratio of embryo volume to grain volume	Vem/Vg
The cavity volume ratio (VRc)	The ratio of cavity volume to grain volume	Vc/Vg
Subcutaneous cavity volume ratio (VRsc)	The ratio of subcutaneous cavity volume to grain volume	Vsc/Vg
Embryo cavity volume ratio (VRemc)	The ratio of embryo cavity volume to grain volume	Vemc/Vg
Endosperm cavity volume ratio (VRenc)	The ratio of endosperm cavity volume to grain volume	Venc/Vg
Density (DE)	The ratio of grain mass to volume	mg/Vg
Sphericity (SP)	The ratio of surface area of the same volume sphere to grain surface area	d/SAg

*d* surface area of same volume sphere

### Method for measuring grain breakage rate

To determine the moisture content of each variety, one hundred kernels were weighed (Wa) and then dried at 85 °C for about 48 h and then weighed again (Wb). The moisture content of the variety was calculated by dividing the difference of weight before and after drying (Wa − Wb) by before drying (Wa) [30]. The moisture contents were 12.4%, 13.4%, 11.5%, 12.5%, 12.8%, and 11.6% for KY3564, M751, DH618, KX9384, LC808, and XY335, respectively.

The grinding method, using milling followed by sieving, was used to measure the grain breakage rate. The method is described by Vyn and Moes [1]. Samples were selected from the middle of the maize ears and were milled using a digital ultrafine grinder (WIGGENS PX-MFC90D, German) fitted with a 2-mm sieve. The test parameters were set as follows: the grain mass was 30 g, the rotation speed was 1200 rpm, and the time was 80 s. A two sieve method was used to sift the granules after milling; a 5 mm-sieve was placed on a 2-mm sieve that was fitted with a receiving pan. The two sets of sieves and pans were stacked on top of each other. After sieving, the maize meal adhering to the bottom of the 5-mm sieve was gently brushed off into the 2-mm sieve and the 5-mm sieve was weighed (W_5mm_). The maize meal adhering to the bottom of 2-mm sieve was brushed off into the receiving pan and the 2-mm sieve was weighed (W_2mm_). The empty receiving pan weight (W_p_), weighed after the sieving and shaking step, was deducted from that of the pan. The breakage rate (BR) was determined according to the equation:

1 $${\text{BR}} = {\text{ W}}_{{\text{p}}} / \left( {{\text{W}}_{5{{\text{mm}}}} + {\text{ W}}_{2{{\text{mm}}}} + {\text{ W}}_{{\text{p}}} } \right)$$

The BR measurements were replicated three times for each variety.

### Data analysis

ANOVA was performed to compare averages for the respective measurements (breakage rate, volume, area, volume ratio, shape size, sphericity, and density). Correlation analyses were performed using SPSS 19.0. Spearman’s rank correlation coefficients were used to test relationships between breakage rates and variable parameters. A stepwise regression was performed by SPSS 19.0 to determine the relationship between the breakage rate and the variable, which is a powerful independent variable selection, and the variable with significant influence on component remained. All variables in the regression were checked to see if any could be removed, using the greater than 5% significance criterion. The process continued until no variables could be added or removed. The remaining variable was used for further analysis. After analyzing the relationship between parameters and breakage rate by stepwise regression, a linear regression equation was established and correlations were examined as well. The path analysis revealed the direct and indirect influence of detected indicators on the dependent variable, which showed the interaction among the parameters. Based on the result of the stepwise regression and correlation analysis, the direct path coefficients were simultaneously obtained, then the indirect path coefficients of each independent variable were completed using correlation analysis × direct path coefficients [31].

## Result

### The breakage rate of the tested varieties

The grain breakage rate is one of the most concerning issues in production. The breakage resistances of the six tested varieties were significantly different (Table 2). There were significant differences between the breakage rates of the six varieties, of which KX3564 was the most vulnerable and XY335 was the most resistant to breakage. This method can be used to distinguish the difference in breakage rate of different varieties.

**Table 2:** Comparison of breakage rates between six grain varieties

Varieties	Breakage rate/%
KY3564	61.9a
M751	57.2b
DH618	56.4b
KX9384	54.6bc
LC808	51.6cd
XY335	49.7d

Mean in the same column followed by different lower case letters indicate a significant difference (P < 0.05)

### Comparison of morphology and internal structure parameters of different varieties

As shown in Table 3, the volumes of grain, endosperm, and embryo for DH618 were significantly higher than other varieties, and the volume was significantly lower for M751 and KX3564 compared to the other varieties. The endosperm and seed coat volume of the six varieties accounted for 86.0–89.3% of the whole grain volume, the embryo accounted for 8.15–10.6% of the grain volume, and the cavity accounted for 2.81–4.92% of the grain volume. The specific surface area is the ratio of the surface area to the grain volume. Grains with a more irregular grain shape had larger specific surface areas. Specific surface area was used to characterize the shape features. The positions of the cavities in the interior of the grains were dispersed, and the shapes and sizes were different leading to the largest specific surface area of the cavity.

**Table 3:** Comparison of structural parameters of different varieties

Variety	Volume/mm^3^							Volume ratio/%						Weight/g
	Grain	Endosperm	Embryo	Cavity	SC	EMC	ENC	Endosperm	Embryo	Cavity	SC	EMC	ENC	Grain
XY335	285.9ab	248.5ab	26.2b	11.2a	3.85ab	3.51a	3.80b	86.9ab	9.22ab	3.84a	1.34ab	1.20a	1.30b	0.346d
M751	280.6b	241.0b	29.8ab	10.3a	4.49ab	1.58a	4.29ab	86.0b	10.55a	3.65a	1.57ab	0.56a	1.53ab	0.356c
DH618	327.8a	282.5a	34.0a	11.0a	3.85ab	3.99a	3.15b	86.2b	10.43a	3.28a	1.20ab	1.14a	0.94b	0.382a
KX3564	283.7b	244.2b	26.3b	13.9a	5.86a	2.22a	5.79ab	86.1b	9.27ab	4.92a	2.10a	0.77a	2.05ab	0.323e
KX9384	305.5ab	272.8ab	24.9b	8.64a	1.35b	2.39a	5.03ab	89.3a	8.15b	2.81a	0.44b	0.79a	1.64ab	0.340d
LC808	295.5ab	251.2ab	30.3ab	14.3a	2.70ab	3.39a	8.20a	85.1b	10.26a	4.82a	0.91ab	1.15a	2.76a	0.364b

Variety	SA/mm^2^				SSA/mm^−1^				Size/mm			AR	DE/g cm^−1^	SP
	Grain	Endosperm	Embryo	Cavity	Grain	Endosperm	Embryo	Cavity	Length	Width	Thickness	Grain	Grain	Grain
XY335	371.0b	699.6a	155.2b	280.3a	1.30a	2.80a	5.92a	26.2a	14.0a	8.05b	4.11b	1.73a	1.278a	0.565a
M751	409.4ab	693.6a	184.6ab	226.3a	1.46a	2.87a	6.30a	21.4a	14.0a	7.85c	4.21b	1.79a	1.198ab	0.510a
DH618	454.6a	788.6a	207.0a	262.6a	1.39a	2.79a	6.09a	24.6a	14.2a	8.62abc	4.84a	1.65ab	1.175ab	0.507a
KX3564	406.3ab	777.3a	175.4b	296.3a	1.44a	3.21a	6.67a	21.7a	14.1a	8.36abc	4.07b	1.69a	1.140b	0.514a
KX9384	429.0ab	722.2a	151.0bc	236.7a	1.41a	2.66a	6.10a	27.4a	12.9b	8.89a	4.41ab	1.46b	1.177ab	0.513a
LC808	404.8ab	808.2a	176.6ab	339.3a	1.37a	3.22a	5.85a	24.8a	13.8a	8.79ab	4.33b	1.58ab	1.233ab	0.530a

SC, EMC, ENC, AR, DE, and SP stand for subcutaneous cavity, embryo cavity, endosperm cavity, aspect ratio, density, and Sphericity respectively. Mean in the same column followed by different lower case letters indicate a significant difference (P < 0.05). All data above was also showed by figure in the Additional file 1

The cavity was divided into three parts to analyze the effect on grain properties. The endosperm cavity volume of KX3564 and LC808 were higher than the subcutaneous and embryo cavities, and their volume ratio was the largest of the six varieties (Table 3). The volume and volume ratio of XY335, M751, and KX3564 were similar between the subcutaneous and endosperm cavities. As expected, the embryo had the least cavity volume and volume ratio among the three parts. In addition, DH618 had the largest subcutaneous cavity volume and proportion among the three parts, while the endosperm cavity volume was significantly smaller than other varieties.

The length, width, thickness, aspect ratio, and sphericity are important parameters of the external morphology, which can reflect grain characteristics of different varieties. As shown in Table 3, the length of the six test varieties was between 12.9 and 14.2 mm, the width was between 7.85 and 8.89 mm, and the thickness was between 4.07 and 4.84 mm. Grain width and thickness were significantly different among the 6 varieties. KX9384 had the shortest length and the longest width and, therefore, the lowest aspect ratio. M751 had the shortest width and the highest aspect ratio value in comparison with other varieties.

The sphericity value reflects the shape characteristics of the grain. The largest and smallest sphericity values of the six varieties were 0.565 and 0.507, respectively. The average weight differed significantly among the six varieties; the highest and lowest weights were 0.382 g and 0.323 g for DH618 and KX3564, respectively.

**Fig. 4:**
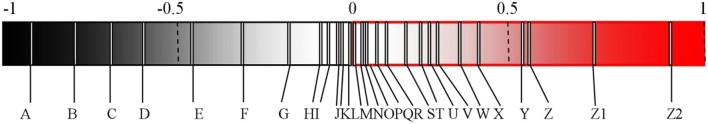
Correlation analysis of grain morphology and structural parameters with breakage rate. From left to right: A: Density (− 0.934**); B: Sphericity (− 0.714*); C: The cavity specific surface area (− 0.628*); D: Embryo cavity volume ratio (− 0.551); E: Embryo cavity volume (− 0.436); F: Grain weight (− 0.371); G: Cavity surface area (− 0.138); H: Endosperm volume ratio (− 0.063); I: Endosperm cavity volume (− 0.049); J: Embryo volume ratio (− 0.015); K: Endosperm volume ratio (− 0.014); L: Endosperm volume (− 0.004). M: Thickness (0.001); N: Width (0.007); O: Embryo volume (0.011); P: Grain volume (0.013); Q: Cavity volume (0.057); R: Aspect ratio (0.087); S: Cavity volume ratio (0.209); T: Length (0.249); U: Endosperm surface area (0.288); V: The Endosperm specific surface area (0.308); W: Embryo surface area (0.374); X: Grain surface area (0.431); Y: Subcutaneous Cavity volume ratio (0.581*); Z: Subcutaneous Cavity volume (0.589*); Z1: Grain specific surface area (0.758*); Z2: Embryo specific surface area (0.927**)

The cavity volume was included in the μCT density measurements. The density was measured using X-ray μCT and significantly influenced the breakage rate (Fig. 4). The grain density of XY335 was significantly higher than that of the other varieties and breakage rate decreased as density increased.

### The correlation analysis of morphology, structural parameters, and the breakage rate based on the X-ray μCT results

Figure 4 shows a correlation analysis of the morphology and structural parameters of grain related to breakage rate. The color from black (negative correlation) to white (no correlation) to red (positive correlation) represents the correlation degree range from − 1 to 1; the color is darker as the correlation increases. The cut-off of − 1 and 1 indicates perfect correlation. The value left of − 0.5 and right of 0.5 represent the parameters that were significantly relevant to the breakage rate. The regression coefficients, from − 0.5 to 0 and from 0 to 0.5, represent negative and positive correlations with breakage rate, respectively, which did not reach significance. The parameters that positively correlated with the breakage rate include specific surface area, three-dimensional size (length, width, and thickness), aspect ratio, cavity volume, surface area (grain, endosperm, and embryo), embryo volume ratio, and cavity volume and its proportion. Grain specific surface area (r = 0.758*), embryo specific surface area (r = 0.927**), subcutaneous cavity volume ratio (0.581*), and subcutaneous cavity volume (0.589*) are significantly and positively correlated with breakage rate.

The parameters that negatively correlated with breakage rate include sphericity, density, volume (grain, endosperm, and embryo), cavity specific surface area, and endosperm volume ratio. The correlation of the cavity specific surface area (− 0.628*) and the grain density (r =  − 0.934**) to breakage rate reached significance. Interestingly, the breakage rate was negatively correlated with sphericity (r = − 0.714*), which indicates that the more spherical a grain, the harder the grain is to break. Meanwhile, the breakage rate was negatively correlated with grain weight, which indicates that heavier kernels are more difficult to break. However, the influence of grain weight on breakage was not significant (r = − 0.370).

### Stepwise regression and path analysis

Stepwise regression had a very strong ability to discriminate all studied shape and structural parameters in terms of the combined effect on the breakage rate. Through stepwise regression analysis, according to partial regression coefficients, equation intercepts, and significance of test results, the following linear regression equation was obtained:

2 $${\text{Y }} = 1.49315 - 0.00012{\text{X}}1 + 0.00830{\text{X}}2 + 0.24457{\text{X}}3 - 0.87871{\text{X}}4, \quad {\text{ R}}^{{2}} = 0.99972$$

X1, X2, X3, and X4 represent the grain specific surface area, subcutaneous cavity volume, sphericity, and density, respectively.

The direct regression coefficients of independent variables X1, X2, X3, and X4 for Y were r_1y_ = − 0.0765, r_2y_ = 0.3016, r_3y_ = 0.1275, and r_4y_ =  − 1.0135, respectively and the significance of the regression coefficients were 0.1491, 0.0048, 0.1099, and 0.0015, respectively. Because the significance of regression coefficients of X1 and X3 were greater than 0.05, stepwise regression was done again. Through stepwise regression, the final linear regression equation was obtained as follows:

3 $${\text{y}} = 1.381 + 0.010{\text{X}}2 - 0.724{\text{X}}4,\quad {\text{ R}}^{{2}} = 0.991$$

The direct correlation coefficients of X2 and X4 to Y were 0.965 and − 0.993 respectively. The significance of the regression coefficients were 0.008 and 0.001 respectively, which were less than 0.05. Equation 3 can be used as a regression equation for correlation factors for the breakage rate.

As shown in Table 4, among the four independent variables with direct effects on breakage rate, the density (X4) had the largest effect, followed by the volume of subcutaneous cavity (X2), and then the sphericity (X3) and the grain specific surface area (X1). Subcutaneous cavity volume and density were the most important factors influencing the breakage rate. In particular, grain density played the most important role of the four major factors. X1 and X3 had indirect effects on breakage rates through the effects on density, with indirect path coefficients of 0.6775 and − 0.8866.

**Table 4:** Direct and indirect path coefficients for principal components

Indicate	DPC	IPC			
		X1	X2	X3	X4
X1	− 0.0765	–	− 0.0651	− 0.1051	0.6775
X2	0.3016	0.0165	–	− 0.0075	0.278
X3	0.1275	0.063	− 0.0178	–	− 0.8866
X4	− 1.0135	0.0511	− 0.0827	0.1115	–

DPC and IPC stand for direct path coefficient and indirect path coefficient respectively

## Discussion

In this study, the three-part volume and its proportion of the grain were calculated using X-ray µCT. The average volume of the six grain varieties was 296 mm^3^ and the cavities ratio accounted for 2.81–4.92% of the grain volume. The endosperm and seed coat volume of the six varieties accounted for 86.0–89.3% and the embryo accounted for 8.15–10.6% of the grain volume (Table 3). The report by Gustin et al. [16] showed that cavities account for up to 13% of the volume of some maize grain. Guelpa [28] demonstrated that the cavities volume ratio for all 16 kernels accounted for 0.24–3.34% of the grain volume, and the soft hybrid (0.96–4.4%) was significantly higher than that of the hard hybrid (0.27–1.51%). These findings suggest that different research materials and determination methods produce different results. KX3564 had the smallest grain volume and the largest cavity proportion. At the same time, KX3564 had the smallest density and the largest breakage rate. Although the cavity volume ratio was positively correlated with the breakage rate, this correlation was not significant but contributed to the high breakage of grain. The cavity mainly exists in the floury endosperm and the grain varieties with larger floury endosperm have more cavities. A lower ratio of vitreous endosperm/floury endosperm indicated that the variety had low-density. Therefore, the breakage rate was higher compared to other varieties.

Based on the data in Fig. 4 and the stepwise regression analysis, the shape (grain specific surface area, sphericity), geometry parameters (subcutaneous cavity volume), and physical characteristics (density) were the main factors influencing the breakage rate. Density and subcutaneous cavity volume were directly relevant to breakage rate and the shape parameters were indirectly related to the breakage rate through the density. Subcutaneous cavity volume and its ratio significantly and positively correlated with breakage rate. However, the size of the endosperm cavity had little effect on the grain breakage rate. In our study, the correlation analysis between breakage rate and grain dimensions indicated that the higher grain breakage rate was associated with longer, wider, thicker kernels. These results agree with previous theoretical and experimental studies [7, 32]. The sphericity value reflects the shape characteristics of the grain, the higher the sphericity, the higher the globularity. The length, width, and thickness of grain affected the breakage rate through the influence on sphericity, which significant negatively correlated with the breakage rate.

Kernel density is highly correlated with other measures of hardness, including milling characteristics [12, 16, 33, 34]. According to the data in Fig. 4, there is a negative correlation between breakage rate and both quality and density; the higher breakage rate of grain accompanies low quality and density. Vyn et al. [1] emphasized that lower grain weight was associated with increased breakage susceptibility but had no significant relationship and breakage-resistant grain tended to be denser than breakage-susceptible grain. The results of this traditional analysis are consistent with our X-ray µCT results. Through stepwise regression and path analysis, we determined the main parameters affecting grain breakage rate. The results indicated that grain density and subcutaneous cavity volume are the two most important factors affecting grain breakage rate among the parameters examined in this paper. Grain density plays a vital role in grain breakage. The accuracy of the density measurement was tested by comparing the estimated kernel density with other measurement methods. We concluded that accurate grain densities were obtained using X-ray μCT technology, which could exclude cavities that would negatively influence results.

The research on maize grain breakage rate was mainly concentrated in the 1960–1990s, which was consistent with the period when mechanical grain harvesting technology was widely popularized [35]. With the development of science and technology, the internal structure information of grain can now be obtained by X-ray µCT technology, which facilitates deep analyses of the factors affecting grain breakage. In the past, maize grain volume, surface area, and sphericity were difficult to acquire. Thus, the impact of these parameters on the breakage rate was difficult to determine. X-ray µCT technology can easily obtain the geometric, morphological, and structural parameters and perform the correlative factor analysis. The objective of this paper was to obtain the main parameters that are related to the breakage rate using X-ray µCT scanning. This information facilitates the management of field production and breeding.

This is a preliminary study on the factors affecting the breakage rate of different varieties using X-ray μCT. The measurement of each variety was made using only three grain samples, which do not entirely reflect the real variety of characteristics. Varieties with large differences in breakage rates should be selected to determinate geometry and morphology parameters from single ears in more detailed future research. A large number of grains will be used to identify the variability of characteristics and analyze the discrepancy source in the breakage rate. Kernel injury during harvest increases significantly when the moisture content is above 20%, but the actual moisture content for harvest is from 20 to 35% grain-moisture range. The moisture content causes negative effects on maize harvest quality and mass. Next, investigations will focus on the effect of grain geometric and morphology parameters on the breakage rate during the dehydration process, because little detailed research has been conducted in this area.

## Conclusion

The relationship between maize grain shape and breakage rate attracted little attention and has been reported rarely in recent years. In this study, X-ray μCT technology provided 28 parameters, including grain size (length, width, thickness, aspect ratio, and sphericity), spatial geometric characteristics (the volume and proportion of the grain, endosperm, seed coat, embryo, and cavity and surface area), grain density, and other characteristic parameters of maize grain. Using X-ray μCT, cavity volumes can also be quantified in three parts (endosperm, embryo, and subcutaneous) and the size and distribution of different parts can be determined. To our knowledge, this is the first report where the cavity volume is divided into three parts and the volume proportions can be obtained. The results show that the following parameters positively correlate with breakage rates: specific surface area, three-dimensional size (length, width, and thickness), aspect ratio, cavity volume, surface area (grain, endosperm, and embryo), embryo volume ratio, and cavity volume and proportions. The parameters negatively correlated with breakage rate include sphericity, density, volume (grain, endosperm, and embryo), cavity specific surface area, and endosperm volume ratio. Correlation and stepwise regression analysis were used to identify the main parameters that influence grain breakage. The results indicate that subcutaneous cavity volume and density are of importance to breakage rates. Other factors (grain specific surface area and sphericity) have indirect effects on grain breakage rate through the density.

## Supplementary information

**Additional file 1.** Additional figures.

## Data Availability

All the maize varieties included in the study are available from the same density on the same plot in Qitai, Xinjiang in 2017. All the parameters acquired are the original data of X-ray CT scanning and the measured data, which were processed according to the test requirements. The data is reliable and the processing is standardized. All generated or analyzed data during this study are included in this published article. This article is not published nor is under publication elsewhere.

## Abbreviations

μCT: micro-computed tomography
SBT: Stein breakage tester
WBT: Wisconsin breakage tester
W: width
L: length
T: thickness
AR: aspect ratio
mg: weight
Vg: grain volume
Ven: endosperm volume
Vem: embryo volume
Vc: cavity volume
Vsc: subcutaneous cavity volume
Vemc: embryo cavity volume
Venc: endosperm cavity volume
SAg: grain surface area
SAen: endosperm surface area
SAem: embryo surface area
SAc: cavity surface area
SSAg: grain specific surface area
SSAen: endosperm specific surface area
SSAem: embryo specific surface area
SSAc: cavity specific surface area
VRen: endosperm volume ratio
VRem: embryo volume ratio
VRc: cavity volume ratio
VRsc: subcutaneous cavity volume ratio
VRemc: embryo cavity volume ratio
VRenc: endosperm cavity volume ratio
DE: density
SP: sphericity
SC: subcutaneous cavity
EMC: embryo cavity
ENC: endosperm cavity
DPC: direct path coefficient
IPC: indirect path coefficient
