# Supplementary material for: Structural parameters for X-ray micro-computed tomography (μCT) and their relationship with the breakage rate of maize varieties
Source: Plant Methods. 2019 Dec 27;15:161. doi: 10.1186/s13007-019-0538-1 (PMC6933881; doi:10.1186/s13007-019-0538-1)
Supplement: Supplementary file 1 — Additional file 1. Additional figures. [file 13007_2019_538_MOESM1_ESM.docx]

| 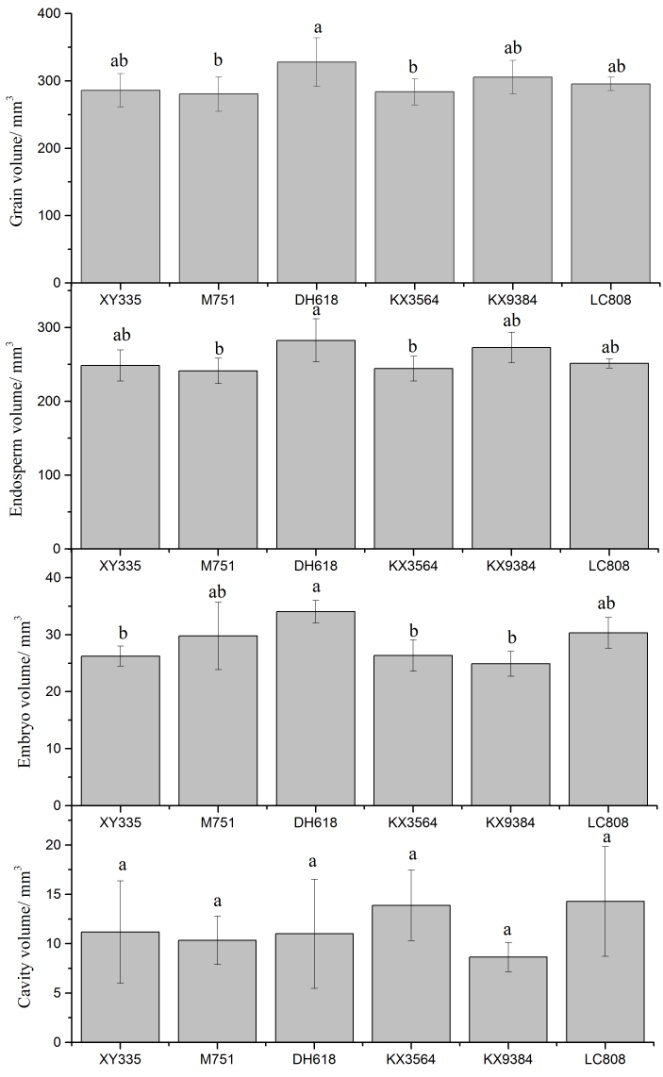 | 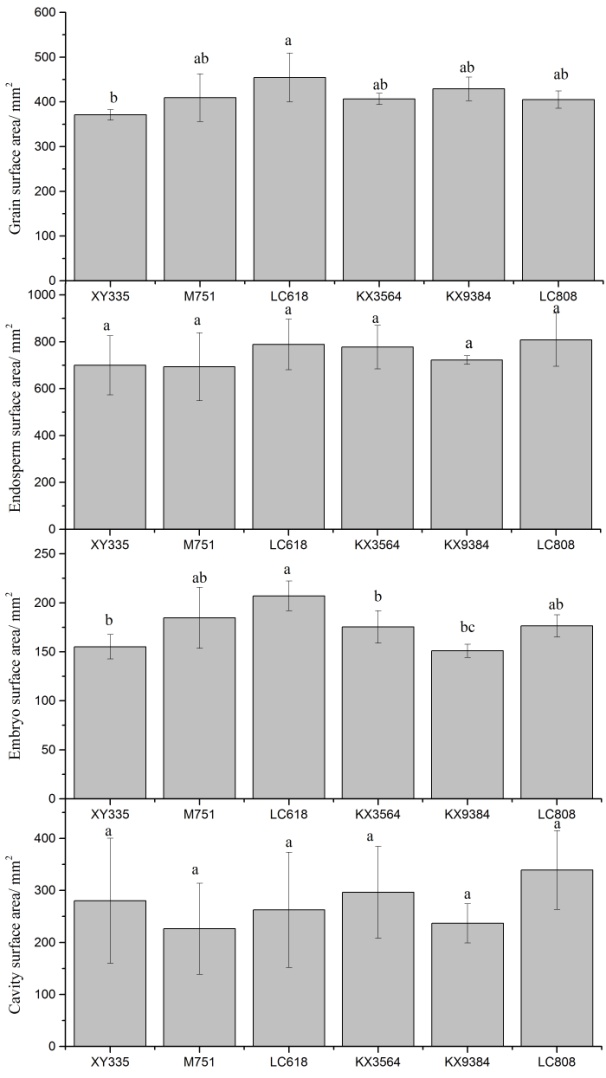 |
| --- | --- |

Additional figure 1 Volume and surface area of grain, endosperm, embryo and cavity of six varieties. The left bar graph is the volume of grain, endosperm, embryo and cavity of six varieties. The right graph is the surface area of grain, endosperm, embryo and cavity of six varieties. Different lower-case letters on the bar chart represent significant difference among the varieties, the same below.

| 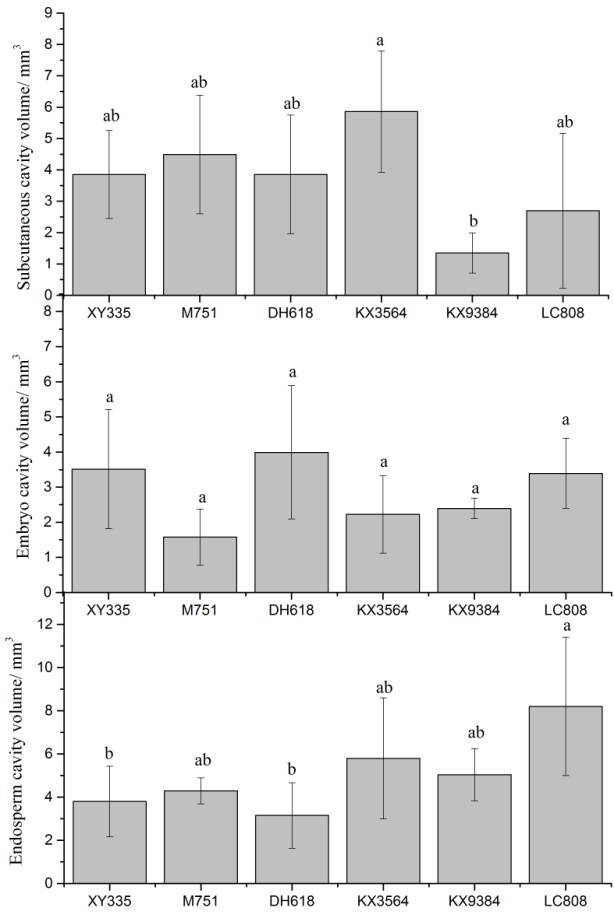 | 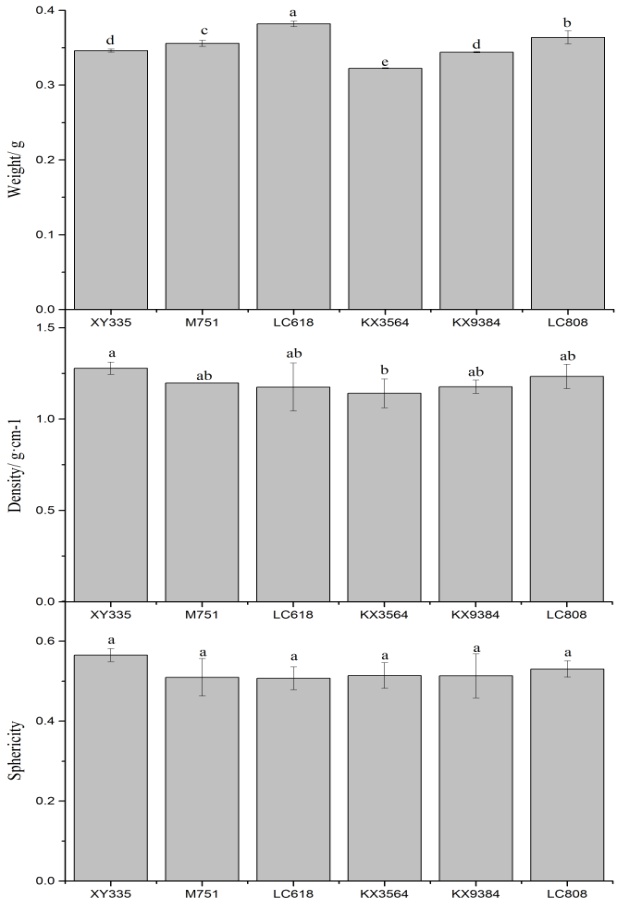 |
| --- | --- |

Additional figure 2 Cavity volume and structural (weight, density and sphericity) parameters of six varieties. The left bar graph is the cavity volume of six varieties. The right bar graph is the structural parameters of six varieties.

|  | |  |
| --- | --- | --- |
| 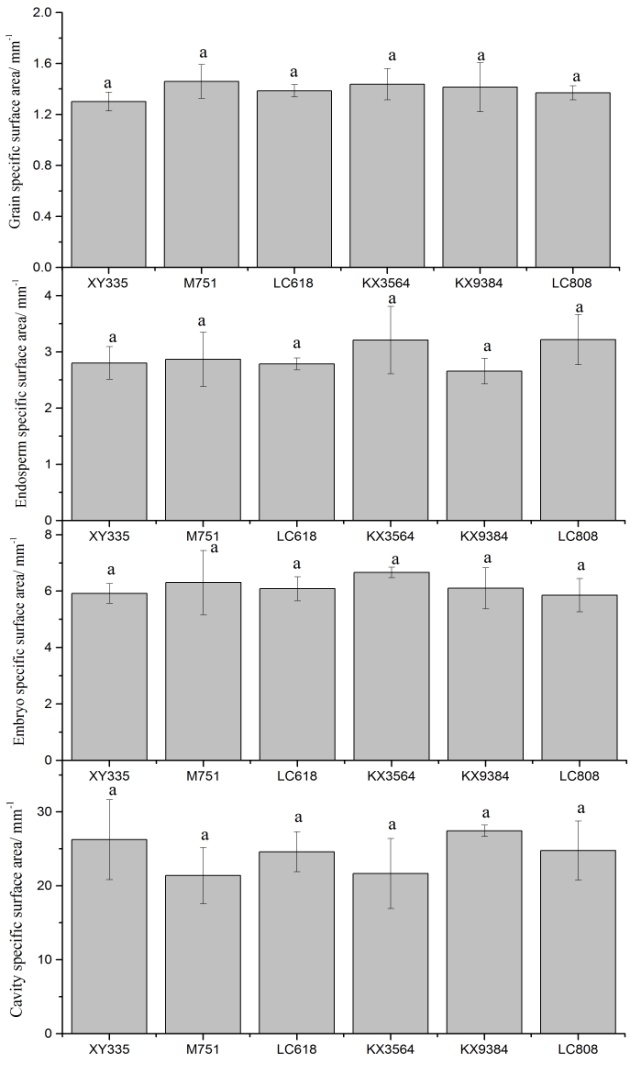 | 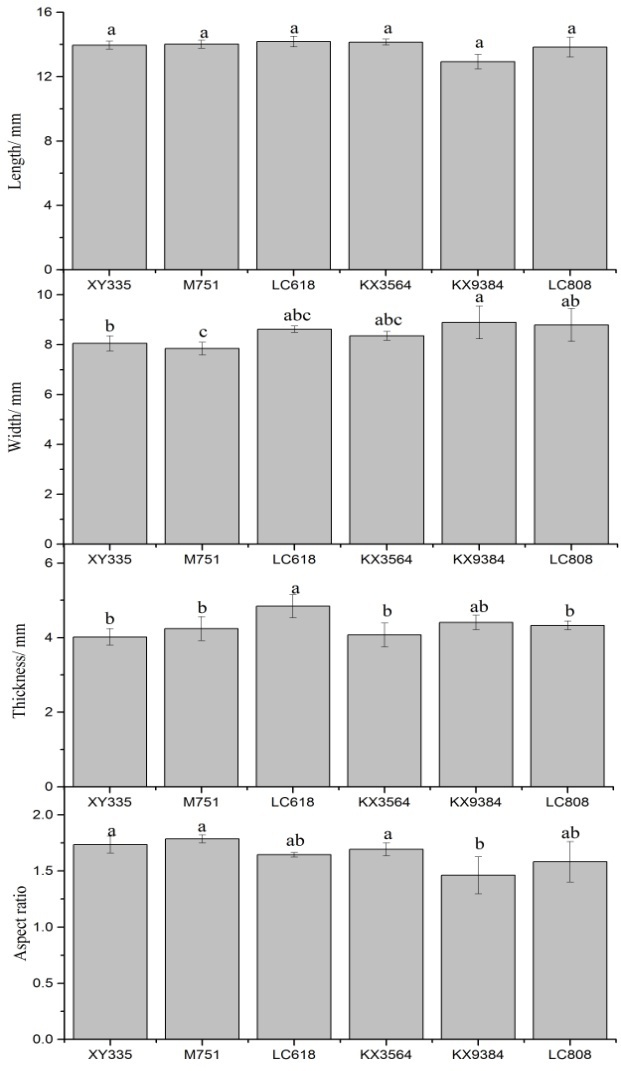 | |

Additional figure 3 The shape parameters of six varieties. The left bar graph is the specific surface area of grain, endosperm embryo and cavity of six varieties. The right bar graph is the size (length, width and thickness) and aspect ratio of grain.


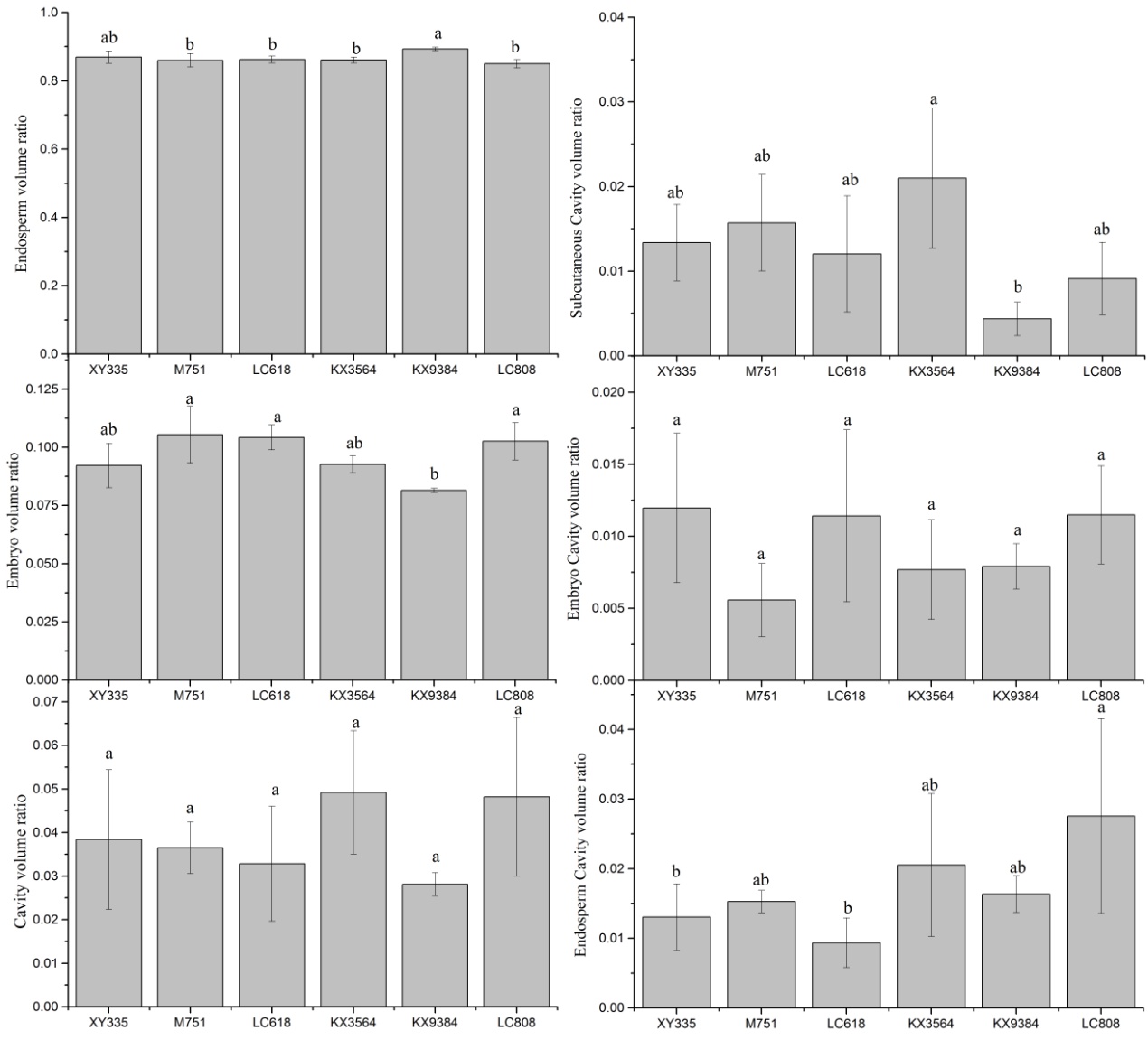


Additional figure 4 The volume ratio of different components of six varieties. The left bar graph is the volume ratio of endosperm, embryo and cavity of six varieties. The right bar graph is the volume ratio of different cavity types of six varieties.
